# A frugal automated contact angle analyser for low-cost surface wettability characterization

**DOI:** 10.1098/rsos.261464

**Published:** 2026-11-04

**Authors:** Rodrigo G. Martins Coelho, Andy Truman, Saravana Kumar Jaganathan

**Affiliations:** ^1^ School of Engineering, University of Leicester, Leicester LE1 7RH, UK; ^2^ Institute of Research and Development, Duy Tan University, Da Nang, Vietnam; ^3^ School of Engineering & Technology, Duy Tan University, Da Nang, Vietnam; ^4^ Biomedical Engineering Research Group, School of Engineering, University of Leicester, Leicester LE1 7RH, UK

**Keywords:** contact angle measurement, surface wettability, Arduino-based instrumentation, image acquisition, surface characterization

## Abstract

Contact angle measurement is a fundamental technique for evaluating surface wettability in materials science, biomedical engineering and coating technologies. However, the high cost of commercial contact angle analysers limits access to surface characterization, particularly in resource-limited laboratories. This study presents a low-cost automated contact angle analyser developed using frugal engineering principles and open-source hardware. The system integrates Arduino-based control, NEMA 17 stepper-motor actuation, automated microdroplet dispensing, smartphone-based optical imaging and a 3D-printed polylactic acid (PLA) framework. A calibrated 330-step dispensing cycle generated reproducible 5 μl water droplets, with fixed optical alignment minimized operator variability. Wettability measurements were performed on polytetrafluoroethylene (PTFE), polyvinyl chloride (PVC) and aluminium substrates and analysed using ImageJ. Mean contact angles were 108.5° for PTFE, 73.9° for PVC and 85.8° for aluminium, closely matching published literature values. One-way analysis of variance (ANOVA) demonstrated significant differences between substrates (*p* < 0.001), with measurement variability of ±2.3° indicated good repeatability. The total fabrication cost was £114.26, representing approximately 99% cost reduction compared with commercial instruments. The proposed platform demonstrates that automated, reproducible surface wettability characterization can be achieved using low-cost, open-source instrumentation, providing an accessible solution for research, education and engineering laboratories while broadening access to advanced materials characterization.

## 1. Introduction

The field of surface science is foundational to modern engineering, acting as a critical gateway for innovations in biotechnology, sustainable energy and advanced manufacturing [1]. Central to this field is the characterization of surface wettability, a property typically quantified by the static contact angle formed at the solid–liquid–gas interface [2]. In the biomedical sector, surface energy dictates the biocompatibility of implants; for instance, the success of a 3D-printed titanium bone scaffold is largely determined by its ability to promote protein adsorption and cellular attachment, both of which are governed by the surface's hydrophilic or hydrophobic nature [3].

However, a profound ‘technological divide’ exists in global research. Although the scientific community agrees on the importance of surface characterization, the infrastructure required to perform it is increasingly consolidated within high-income institutions. Commercial goniometers and contact angle analysers are sophisticated instruments that utilize high-speed imaging and automated dispensing to ensure repeatability. Yet, these systems come with a prohibitive price tag, typically ranging from £11 000 to £22 000 [4,5]. For universities and independent research centres in low-resource country settings, such capital expenditure is often impossible, creating a cycle of ‘research poverty’ where scientists in developing nations are excluded from contributing to high-impact materials science owing to a lack of basic analytical tools.

To circumvent these financial barriers, a movement towards ‘frugal innovation’ has emerged. Researchers have traditionally utilized DIY ‘hacks’, such as using basic manual syringes, improvised lighting and smartphone cameras [6,7]. Although these efforts are commendable for their cost-efficiency, they introduce significant technical trade-offs. Variations in droplet volume, contact line positioning and image acquisition parameters can influence contact angle determination, highlighting the importance of automated dispensing and imaging systems for improving measurement repeatability [8]. Furthermore, manual rigs lack the structural rigidity required for precise camera alignment and focus, which often results in parallax errors and poor image quality [9].

There is, therefore, a critical need for a paradigm shift from manual frugal rigs to automated frugal instrumentation [10,11]. By replacing proprietary sensors and high-cost mechanical stages with open-source Nema 17 stepper motors and biodegradable polylactic acid (PLA)-printed components, it is theoretically possible to mechanize the analytical process without the industrial price tag [12,13]. Recent reviews have extensively demonstrated how open-source hardware frameworks democratize access to complex scientific instrumentation, ensuring high analytical reproducibility while mitigating prohibitive commercial costs [14,15].

The theoretical hypothesis of this research is that ‘frugal automation’ can achieve the mechanical stability and volumetric precision of commercial systems. The primary aim of this study is to develop and validate a fully automated, low-cost contact angle analyser specifically optimized for biomedical and engineering research in low-resource settings. This work seeks to provide a replicable, open-source blueprint that empowers researchers to conduct high-fidelity surface science at a fraction of the traditional cost.

## 2. Material and methods

### 2.1. System design and fabrication

The automated contact angle analyser was developed following principles of open-source hardware to enable high-precision surface characterization at significantly reduced cost [16]. The system architecture consists of a three-axis motorized framework designed to minimize user-induced variability in droplet volume, camera alignment and focal positioning. Actuation was achieved using Nema 17 stepper motors (17HS4401S), providing 43 N cm holding torque for stable positioning. Structural components were fabricated using additively manufactured PLA. To demonstrate the financial viability of this frugal architecture, a complete itemized breakdown of the prototype is listed in table 1, confirming a total build cost of £114.26.

**Table 1.** Bill of materials for the automated contact angle analyser.

bill of materials		
quantity	description	cost
1	CNC Shield, Arduino, stepper drivers	£17.99
1	3 pcs Nema 17 stepper motor	£24.99
1	2 sets 100 and 8 mm T8 lead screw set	£10.99
2	lead screw nut housing	£6.99
1	Trajan SGE 003000 microvolume 25 µl	£35.31
1	PLA 3D printing filament	£17.99
total		£114.26

### 2.2. Automated microdispensing and control system

A motorized fluid handling module was developed to ensure precise and repeatable droplet generation. The system incorporates a microvolume syringe [17], with the plunger actuated via a lead screw-driven *z*-axis mechanism. The system was controlled using an Arduino Uno Rev 3 microcontroller. Control logic was implemented in C++ using discrete step-counting to ensure repeatability. Calibration was performed empirically via iterative actuation and visual verification against the volumetric gradations of the microvolume syringe. Through this process, a 330-step dispensing cycle was established to extrude a nominal 5 µl droplet.

### 2.3. Optical set-up and image acquisition

Image capture was performed using a high-resolution smartphone camera equipped with a 5× optical telephoto lens to reduce optical distortion. The device was mounted on a motorized gimbal, allowing controlled adjustment along the *x*-axis for focus and the *y*-axis for tilt alignment. This configuration ensured that the optical axis remained parallel to the sample surface, which is critical for accurate baseline detection. Captured images were transferred digitally for subsequent processing.

### 2.4. Wettability measurement and statistical analysis

Image analysis was conducted using the open-source software ImageJ [18]. To ensure standardized processing, images were rotated 180° and converted to 8-bit greyscale to enhance contrast between the droplet and substrate. The contact angle was determined using a manual sessile drop method [19], where the droplet profile and baseline were defined using the Contact Angle plugin [20,21].

To evaluate system performance, wettability measurements were conducted on three reference substrates: Polyvinyl chloride (PVC), aluminium and polytetrafluoroethylene (PTFE). To establish a baseline on unmodified commercial surfaces, all materials were tested in their as-received condition directly from the packaging, without the application of cleaning solvents, deionized water washing or plasma/UV treatments. Distilled water droplets of 5 µl were used for all tests. Ten independent measurements were performed for each material to assess repeatability and statistical reliability, enabling evaluation of both measurement precision and the system's ability to distinguish between hydrophilic and hydrophobic surfaces [22]. This methodical approach provides a rigorous basis for comparing the developed frugal system against established laboratory standards. Measurements were conducted in a standard laboratory environment without active climate control. Ambient temperature and relative humidity were not recorded or regulated during testing, reflecting the anticipated real-world operational conditions for frugal instrumentation deployed in low-resource settings.

## 3. Results

### 3.1. System realization and operational performance

The frugally engineered contact angle analyser was successfully fabricated and operated as a fully integrated three-axis automated system. The combination of stepper-motor-driven actuation and 3D-printed structural components enabled stable and repeatable positioning of the dispensing unit and imaging system. The mechanical framework demonstrated sufficient rigidity during operation, with no significant structural deformation observed during testing. The Arduino-based control system provided smooth and synchronized motion across all axes, confirming the effectiveness of the step-counting logic for positional accuracy in the absence of closed-loop feedback. The automated syringe mechanism functioned reliably, enabling controlled extrusion and retraction of liquid without manual intervention (figure 1).

**Figure 1.**
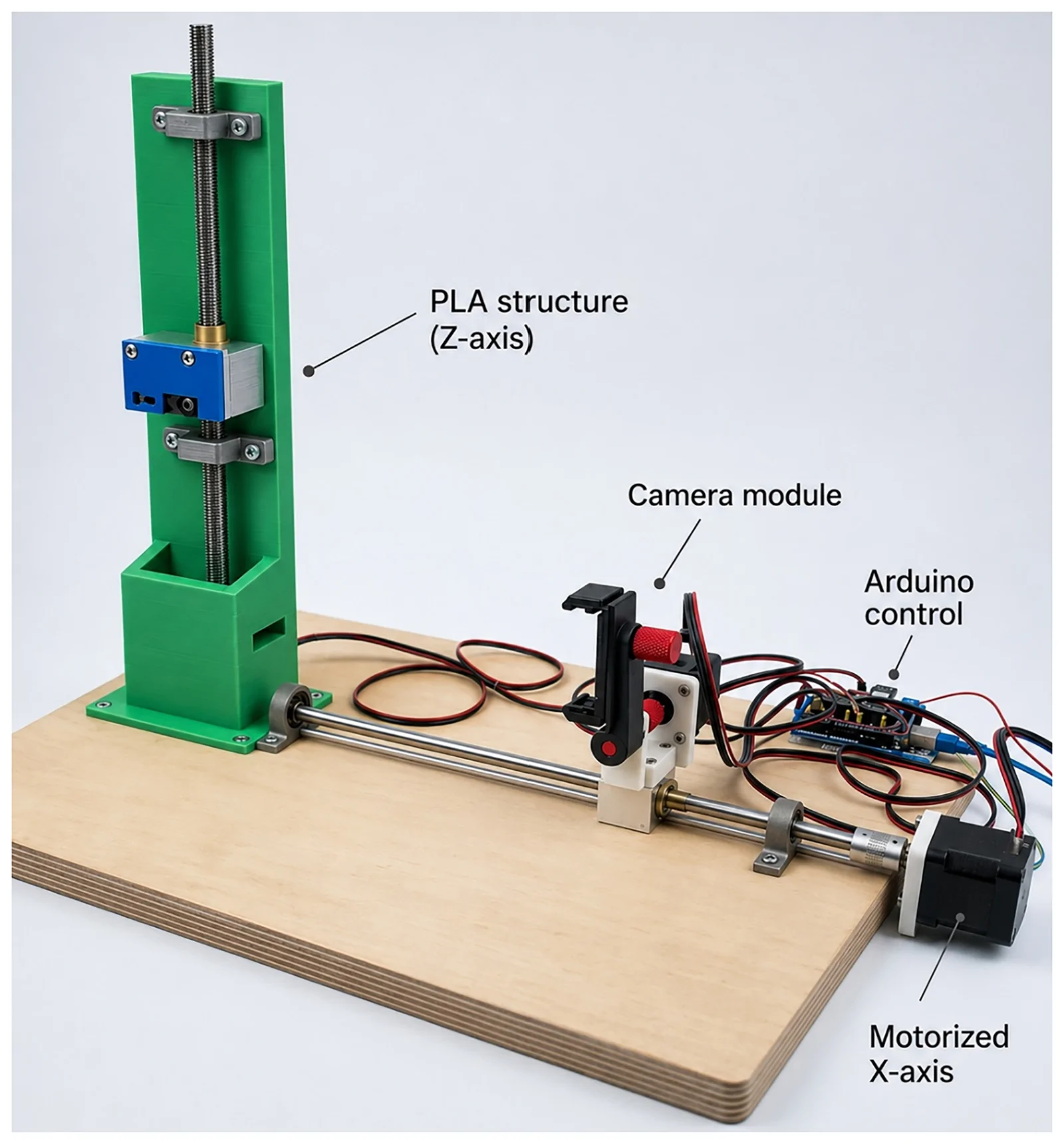
Arduino-based motorized contact angle measurement system. Photograph of the custom-built Arduino-based contact angle measurement system used for automated droplet deposition and image acquisition. The apparatus comprises a three-axis motorized positioning framework actuated by Nema 17 stepper motors (17HS4401S), a camera module for droplet imaging and Arduino-based control electronics. Structural components were fabricated from additively manufactured PLA.

### 3.2. Precision of microdispensing and optical acquisition

The dispensing system consistently produced sessile droplets with a nominal volume of 5 µl, as defined by the calibrated step input of the *z*-axis. This ensured uniform droplet generation across all trials and minimized variability associated with manual pipetting. The integration of the smartphone-based optical system allowed high-resolution image capture of the droplet profile. The motorized positioning enabled repeatable alignment and focal adjustment; however, minor inconsistencies in camera alignment introduced slight optical distortions, which contributed to measurement variability.

### 3.3. Contact angle measurements and statistical analysis

The measured contact angle for PTFE was 108.5° ± 5.6°, which is consistent with the hydrophobic behaviour of PTFE and falls within the range of 108°–110° reported in the literature [23,24]. PVC exhibited a mean contact angle of 73.9° ± 6.8°, which is lower than the 80°–85° values commonly reported for smooth PVC surfaces but may be attributed to differences in surface roughness, film morphology and substrate effects [25,26]. Aluminium showed a mean contact angle of 85.8° ± 6.9°, which is slightly higher than values reported for highly polished aluminium surfaces but remains within the range observed for commercially processed or textured aluminium substrates [27,28]. Overall, the measured contact angles are in reasonable agreement with published values, confirming the capability of the developed system to accurately distinguish surfaces with different wettability characteristics. The mean contact angle and corresponding standard deviation for each substrate are presented in figure 2. Note that the measured contact angles for PVC and aluminium are probably influenced by surface topography. According to the Wenzel wetting model, surface roughness such as machining marks on aluminium or extrusion textures on PVC increases the effective surface area. For materials with inherent contact angles below 90°, this roughness amplifies hydrophilicity, lowering the apparent contact angle [1]. This topographical effect may partially account for the minor deviations observed compared with ideally smooth reference surfaces.

**Figure 2.**
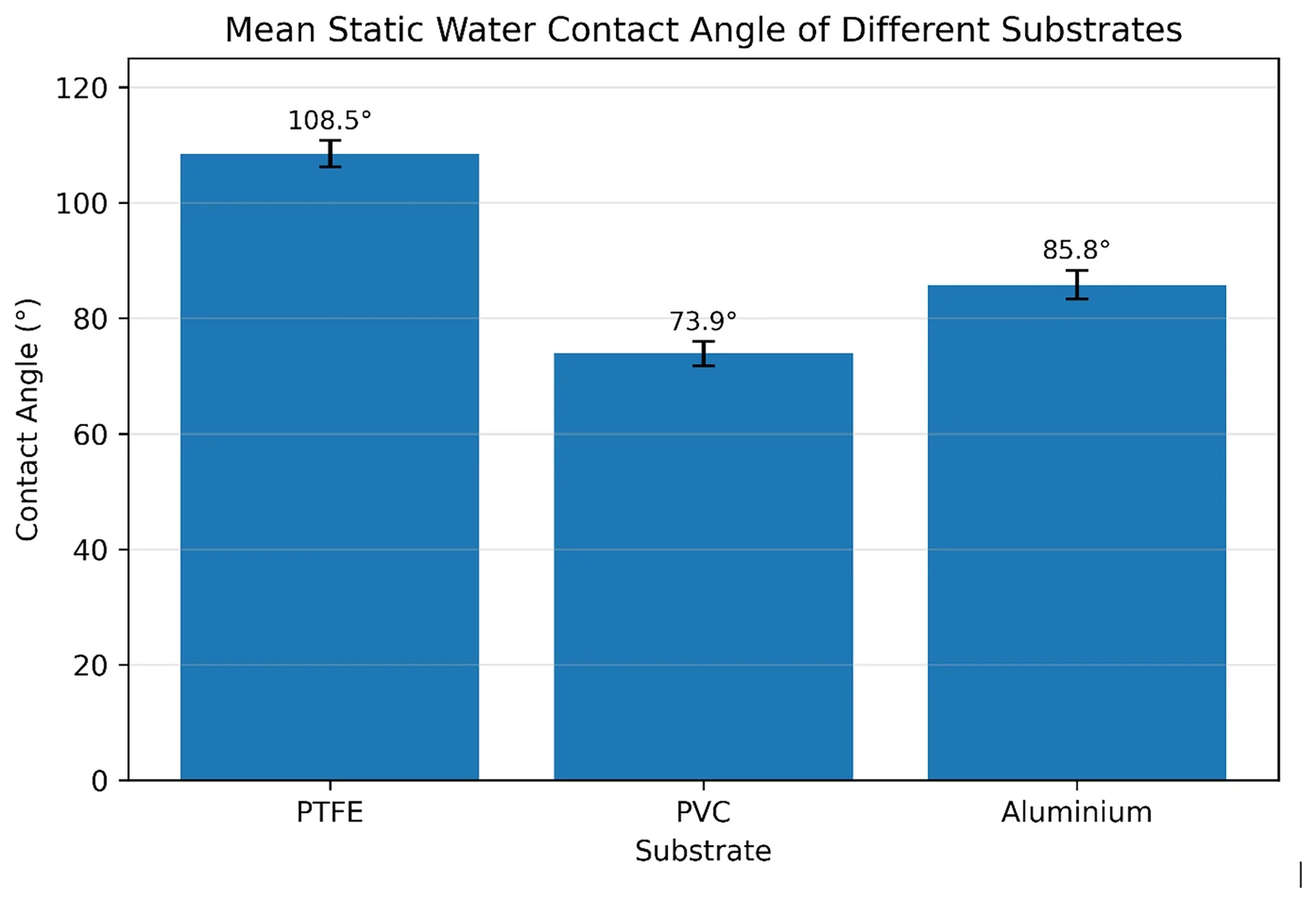
Mean static water contact angle of PTFE, PVC and aluminium substrates. Comparison of the mean static water contact angles measured on PTFE, PVC and aluminium substrates (*n* = 10). PTFE exhibited the highest contact angle (108.5° ± 5.6°), indicating the most hydrophobic surface, whereas PVC showed the lowest contact angle (73.9° ± 6.8°), indicating greater wettability. Aluminium displayed an intermediate contact angle (85.8° ± 6.9°). Differences among the three materials were statistically significant (one-way ANOVA, *p* < 0.001). Error bars represent 1 s.d. from the mean.

To measure the system's mechanical variability independently from natural variations across the materials (such as surface roughness), new 5 µl droplets were repeatedly dispensed and measured on a single, uniform area. The standard deviation across these repeated cycles was ±2.3°, defining the baseline repeatability of the automated dispensing and imaging system. This magnitude of mechanical tool variability was small relative to the observed inter-substrate differences, suggesting that measurement noise had limited influence on the observed separation between materials.

The variability in measurements differed across substrates, with PTFE showing lower dispersion compared with PVC and aluminium. PTFE consistently exhibited contact angles greater than 90°, consistent with hydrophobic behaviour, whereas both PVC and aluminium showed contact angles below 90°, consistent with hydrophilic behaviour. A one-way analysis of variance (ANOVA) revealed a statistically significant difference in mean contact angles among the three materials (F_2,27_ = 74.04, *p* < 0.001, *η*² = 0.85). Post hoc Tukey's honestly significant difference testing confirmed that all pairwise comparisons between substrates were statistically significant (PTFE versus PVC: *p* < 0.001; PTFE versus aluminium: *p* < 0.001; PVC versus aluminium: *p* < 0.001). The magnitude of mechanical tool variability (±2.3°) was small relative to the observed inter-substrate differences, suggesting that measurement noise had limited influence on the observed separation between materials.

## 4. Discussion

The development of the automated contact angle analyser represents a significant departure from conventional paradigms of laboratory instrumentation, demonstrating that high-precision scientific capability can be approached through frugal automation without compromising data integrity [12]. With a total hardware cost of £114.26, the system achieves an approximate 99% cost reduction relative to conventional commercial goniometers, which typically range between £11 000 and £22 000 [4,5]. This substantial reduction is not only an economic achievement but also a methodological rethinking of how scientific infrastructure can be designed, deployed and distributed. By integrating low-cost embedded electronics with mechanically stabilized automation, the system maintains measurement fidelity while improving accessibility of surface science instrumentation.

From a sustainability perspective, the platform embodies key principles of circular design and low-impact engineering. The use of PLA, an industrially compostable polymer, for structural components reduces dependence on petroleum-derived engineering plastics and aligns with broader efforts to minimize laboratory-based material waste. Coupled with a low-voltage operational profile, the system reduces both embodied and operational energy demands, thereby contributing to reduced carbon intensity in experimental workflows. These design choices collectively align with UN Sustainable Development Goal 9, demonstrating that innovation in scientific infrastructure can support industrial capability, environmental responsibility and technological inclusivity. Importantly, the system challenges the assumption that precision instrumentation must be inherently resource-intensive, instead suggesting that sustainability and performance can be co-optimized rather than treated as competing objectives.

A central contribution of this work lies in addressing a long-standing limitation in low-cost experimental science: the reproducibility gap associated with manual or semi-manual measurement systems. Traditional frugal set-ups for contact angle measurement often rely on handheld droplet deposition and smartphone-based imaging, both of which introduce significant operator-dependent variability. These include inconsistencies in droplet volume and release height, as well as optical misalignment arising from non-fixed imaging geometries [6,9]. The automated system mitigates these limitations through mechanically constrained dispensing and motion-controlled actuation, achieving highly repeatable droplet generation with a controlled 330-step actuation cycle for 5 µl delivery. By reducing operator involvement in the most variability-prone stages of the protocol, the system improves repeatability and reproducibility.

Equally important is the reduction of geometric and optical inconsistencies that are typically overlooked in low-cost experimental rigs. Fixed optical alignment and controlled *x*-axis focal adjustment ensure that droplet profiles are captured under consistent imaging conditions, reducing parallax distortion and perspective-based measurement errors. Controlled imaging conditions are important for obtaining reliable measurements of advancing and receding contact angles, particularly when assessing droplet behaviour on solid surfaces. This transition from manually assembled ‘hackable’ systems to structurally constrained automation represents a step towards ensuring that low-cost instrumentation can meet the rigour required for publishable and comparable scientific data. The resulting measurements support the view that precision is strongly influenced by the control of experimental variables through engineering design, rather than solely by capital-intensive infrastructure.

The broader implications of this system are particularly significant for research environments in low- and middle-income countries, where access to advanced materials characterization tools is often limited by prohibitive capital costs [10]. In such contexts, the absence of surface science instrumentation constrains not only academic research output but also local innovation in fields such as biomaterials, agriculture and environmental engineering. By leveraging widely available microcontroller platforms such as the Arduino Uno and incorporating open-source design principles [13], the present system lowers the entry barrier for establishing functional surface science laboratories. This enables local researchers to directly engage in materials optimization studies, such as hydrophobic coating development for water harvesting systems or surface modification of filtration membranes for improved water purification.

The experimental validation across multiple substrate classes further demonstrates the system's applicability as a generalized tool for materials characterization. The measured contact angles for PTFE (108.5°), PVC (73.9°) and aluminium (85.8°) are consistent with established literature values [23,25,28], supporting the reliability of the automated approach. These materials are widely used in engineering and biomedical applications. PTFE and PVC, for example, are widely employed in medical tubing and implant housings. In these biomedical contexts, surface wettability is a primary driver of biological integration; studies have consistently demonstrated that moderate surface hydrophilicity dictates protein adsorption profiles, which in turn optimally support integrin-mediated cell attachment, spreading and proliferation on biomaterials [3,29]. Conversely, highly hydrophobic surfaces often drive adverse bacterial colonization and biofouling. Similarly, aluminium surfaces are important in structural engineering and biomedical device casings, where wettability affects coating adhesion, corrosion resistance and long-term durability. The ability of the system to distinguish between hydrophobic and hydrophilic regimes across these substrates highlights its potential utility in both industrial and biomedical contexts.

The observed statistical consistency, with a standard deviation of approximately ±2.3°, further supports the robustness of the system. This level of precision is notable given the low-cost and lightweight nature of the mechanical framework, suggesting that measurement stability is primarily governed by system design and control of experimental conditions rather than component cost alone. Although minor limitations are acknowledged, particularly the susceptibility of the PLA-based structure to external vibrations, these effects are mitigated through controlled laboratory placement and could be further addressed through future material upgrades or hybrid structural reinforcement. Importantly, these limitations do not undermine the validity of the measurement methodology but instead define pathways for iterative improvement.

The modularity of the system represents one of its most significant strengths from a scalability perspective. The Arduino-based control architecture enables straightforward integration of additional functionalities, including temperature-controlled environmental stages, automated droplet shape recognition using computer vision algorithms and machine-learning-assisted contact angle extraction. Such enhancements would further reduce user dependency while increasing throughput and analytical sophistication. More broadly, the system establishes a foundation for a distributed model of scientific instrumentation, where advanced measurement capabilities are no longer confined to centralized, high-cost facilities but can instead be deployed across geographically and economically diverse research environments.

Collectively, this work demonstrates that high-precision surface science instrumentation can be reimagined through the principles of frugal automation, sustainability and open design. By partially decoupling scientific capability from industrial cost structures, the system provides a pathway towards more equitable global access to advanced research tools. In doing so, it contributes not only to methodological innovation in contact angle measurement but also to a broader reconfiguration of how scientific infrastructure is conceived within the context of global challenges, resource constraints and sustainable development imperatives.

## 5. Conclusion

This study (figure 3) demonstrates that a fully automated, low-cost contact angle measurement system can achieve high scientific fidelity at a total build cost of £114.26, representing an approximately 99% reduction compared with conventional commercial instruments. The system delivered consistent and reliable measurements across benchmark substrates, with experimentally validated contact angles of 108.5° (PTFE), 73.9° (PVC) and 85.8° (aluminium), and maintained a low variability of ±2.3°, confirming strong repeatability despite its frugal design. By integrating controlled droplet dispensing, fixed optical alignment and automated imaging, the platform effectively eliminates key sources of operator-dependent error common in manual low-cost set-ups, thereby bridging the gap between affordability and precision in surface wettability analysis.

**Figure 3.**
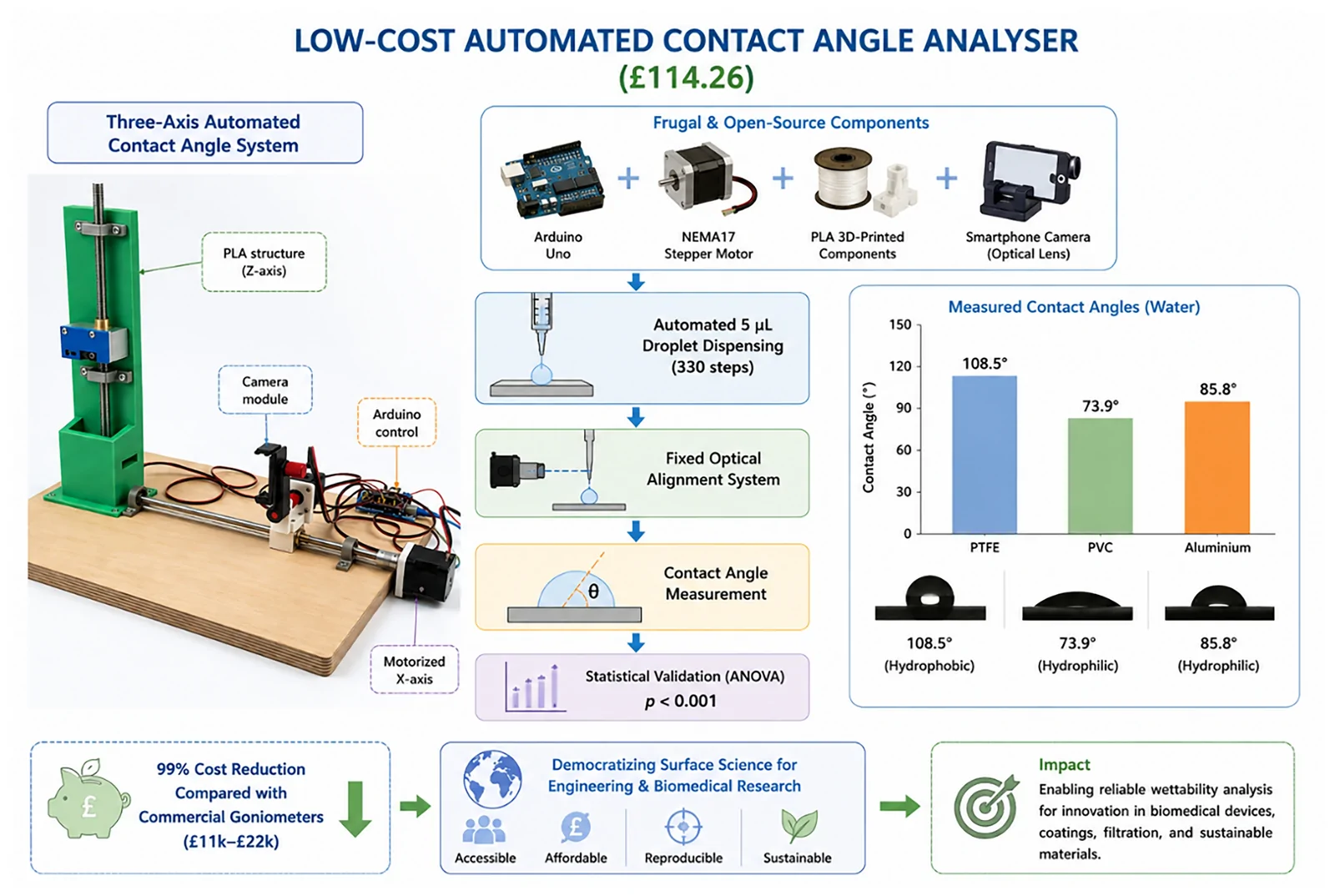
Graphical summary of the proposed low-cost automated contact angle analyser and its key findings. The figure highlights the frugal, open-source design, automated operational workflow and experimental validation of the developed system. The analyser integrates Arduino-based control, stepper-motor-driven droplet dispensing, smartphone-based imaging and 3D-printed components to achieve reliable and reproducible contact angle measurements at a total fabrication cost of £114.26. Validation on PTFE, PVC and aluminium substrates demonstrated good agreement with published literature values and significant differences in wettability (*p* < 0.001), while achieving an approximate 99% reduction in cost compared with commercial contact angle analysers. The platform provides an accessible, affordable and reproducible solution for surface wettability characterization, supporting broader adoption of advanced materials characterization in engineering, biomedical research and resource-limited laboratories. This figure was created using ChatGPT (OpenAI) as a generative AI tool. The authors reviewed, verified and edited all text and graphical elements to ensure their accuracy and consistency with the study.

The significance of this work lies in its demonstration that high-quality materials characterization can be decentralized without reliance on expensive proprietary infrastructure. This positions the system as a practical enabler for laboratories in resource-constrained settings, supporting local innovation in biomedical devices, coatings, filtration systems and sustainable materials development. Furthermore, the use of open-source electronics and compostable structural materials aligns the platform with sustainable engineering principles and scalable scientific democratization.

Future work will focus on improving structural stability through enhanced vibration isolation and alternative lightweight composite materials, alongside the integration of real-time computer vision algorithms for fully automated droplet edge detection and contact angle computation. Additional developments may include environmental control modules (temperature and humidity regulation) and machine learning-based calibration to further enhance accuracy and adaptability across diverse liquid–solid systems. Collectively, these advancements will move the system towards a fully autonomous, field-deployable platform for next-generation frugal surface science, proving that rigorous scientific inquiry is not bound by capital expenditure but driven by engineering ingenuity.

## Data Availability

All data supporting the findings of this study are contained within the article. No additional datasets are associated with this article.
